# Towards better guidance on caseload thresholds to promote positive tuberculosis treatment outcomes: a cohort study

**DOI:** 10.1186/s12916-016-0592-8

**Published:** 2016-03-23

**Authors:** Helen R. Stagg, Ibrahim Abubakar, James Brown, Maeve K. Lalor, H. Lucy Thomas, Tehreem Mohiyuddin, Debora Pedrazzoli, Corinne S. Merle

**Affiliations:** Research Department of Infection and Population Health, University College London, London, UK; Centre for Respiratory Medicine, Royal Free London NHS Foundation Trust, London, UK; Division of Medicine, University College London, London, UK; Tuberculosis Section, Respiratory Diseases Department, Public Health England, London, UK; CMMID, London School of Hygiene and Tropical Medicine, London, UK; TB Centre, London School of Hygiene and Tropical Medicine, London, UK; TB Modelling Group, London School of Hygiene and Tropical Medicine, London, UK; UNICEF/UNDP/World Bank/WHO Special Programme for Research and Training in Tropical Disease (TDR), Geneva, Switzerland; London School of Hygiene and Tropical Medicine, London, UK

**Keywords:** Caseload, Treatment outcomes, Tuberculosis, Workload

## Abstract

**Background:**

In low-incidence countries, clinical experience of tuberculosis is becoming more limited, with potential consequences for patient outcomes. In 2007, the Department of Health released a guidance ‘toolkit’ recommending that tuberculosis patients in England should not be solely managed by clinicians who see fewer than 10 cases per year. This caseload threshold was established to try to improve treatment outcomes and reduce transmission, but was not evidence based. We aimed to assess the association between clinician or hospital caseload and treatment outcomes, as well as the relative suitability of making recommendations using each caseload parameter.

**Methods:**

Demographic and clinical data for tuberculosis cases in England notified to Public Health England’s Enhanced Tuberculosis Surveillance system between 2003 and 2012 were extracted. Mean clinician and hospital caseload over the past 3 years were calculated and treatment outcomes grouped into good/neutral and unfavourable. Caseloads over time and their relationship with outcomes were described and analysed using random effects logistic regression, adjusted for clustering.

**Results:**

In a fully adjusted multivariable model (34,707 cases)there was very strong evidence that management of tuberculosis by clinicians with fewer than 10 cases per year was associated with greater odds of an unfavourable outcome compared to clinicians who managed greater numbers of cases (cluster-specific odds ratio, 1.14; 95 % confidence interval, 1.05–1.25; *P* = 0.002). The relationship between hospital caseload and treatment outcomes was more complex and modified by a patient’s place of birth and ethnicity. The clinician caseload association held after adjustment for hospital caseload and when the clinician caseload threshold was reduced down to one.

**Conclusions:**

Despite the relative ease of making recommendations at the hospital level and the greater reliability of recorded hospital versus named clinician, our results suggest that clinician caseload thresholds are more suitable for clinical guidance. The current recommended clinician caseload threshold is functional. Sensitivity analyses reducing the threshold indicated that clinical experience is pertinent even at very low average caseloads, which is encouraging for low burden settings.

**Electronic supplementary material:**

The online version of this article (doi:10.1186/s12916-016-0592-8) contains supplementary material, which is available to authorized users.

## Background

In 2014, 6,520 cases of tuberculosis (TB) were notified in England [1]. Despite a decline in such case numbers, they are still described as ‘unacceptably high’ by Public Health England (PHE) [1]. In other areas of health, diseases that require specialist care but where overall case numbers are not substantial have had their clinical services centralised. The justification for such centralisation is based on evidence suggesting that outcomes and survival can be improved by the treatment and management of patients by ‘high volume’ clinical specialists and hospitals, e.g. for various cancers [2, 3]. Along similar lines, in 2007, the Department of Health’s TB guidance toolkit (hereafter referred to as the ‘toolkit’) for England advised that: “[if TB] *is confirmed, the patient is best managed by, or in conjunction with, a clinician (a respiratory physician or appropriately trained infectious disease physician) who sees at least 10 confirmed cases per year*” [4]. This caseload threshold was established to improve treatment outcomes and thus reduce transmission, but was not evidence based.

At the time of writing, only one publication could be found looking at the association between clinician caseload and TB case outcomes. Gardam et al. [5] suggested that management by an ‘experienced’ clinician (individuals who saw an average of one case or more a year) had a protective association on the likelihood of death among foreign-born TB cases in Ottawa 1999–2002 (hazard ratio, 0.41; 95 % confidence interval (CI), 0.22–0.77). A second option for recommending caseload thresholds is to do so by treatment centre. Cegolon et al. [6] and Lake et al. [7] examined the likelihood of treatment completion among English and Welsh TB cases in 2001–2006 and London TB cases in 2003–2006, respectively, given TB centre caseload. Cegolon et al.’s [6] study, stratified by place of birth and age, found a disadvantageous association between smaller treatment centres (caseloads of 26 and under) and treatment completion in individuals less than 45 years old born outside the UK. Lake et al. [7] suggested that centres with larger caseloads (130 or more cases) are associated with a lower likelihood of treatment failure.

Using PHE notification data we sought to examine the association between clinician and hospital caseload and treatment outcomes, as well as discuss the relative suitability of each method of recommending caseload thresholds.

## Methods

### Study population

All TB cases notified in England to PHE’s Enhanced TB Surveillance System (ETS) from the 1st January, 2003, to the 31st December, 2012, with an expected treatment duration of 12 months or less were extracted, including their associated clinical, demographic, and laboratory data. Rifampicin (RIF)-resistant cases, non-culture confirmed cases treated as multidrug resistant, and cases with central nervous system, spinal, cryptic disseminated, or miliary disease were excluded due to having a longer expected duration of treatment [1]. Cases diagnosed post-mortem were excluded as their deaths could not have been influenced by the clinician or hospital responsible for their TB treatment.

### Clinician caseload

The named medical doctor (hereafter referred to as ‘clinician’) managing each case is entered at the hospital into the ETS as free text. This field was cleaned and grouped to assign a code for each unique clinician. Where two clinicians were named, alternative groupings were created, as well as a new variable to designate shared clinical management (present or absent/not recorded).

Additionally, on a subset of 10 hospitals in different parts of England (south west, south, London) that accounted for approximately 15 % of overall cases, the clinician name field was further checked and cleaned by one of the authors (who had worked in these hospitals) and classified according to staff type. Managing clinician was restricted here to respiratory and infectious disease specialists. This was felt to be necessary because a review of the clinician field by this author suggested that the named individual was sometimes a caregiver other than the lead consultant (e.g. TB nurse or other medical doctor). This subset of data was reserved for the sensitivity analysis.

The toolkit provides no guidance as to the timeframe over which a clinician’s caseload should be calculated. A pragmatic decision was thus made to calculate mean caseload over the preceding 3 years. This was then grouped in the following ways: (1) above and below the caseload threshold of 10 and (2) post hoc groupings as described in the sensitivity analysis. If two clinicians were named alternative caseload groups were also generated, with the default for analysis to conservatively take the clinician with the highest caseload. Caseload was also recalculated for the 10 hospital subset.

### Hospital caseload

The hospital recorded on ETS usually represents the last one where the patient was known to have been treated. Hospital caseload was calculated in the same way as clinician caseload. In the absence of toolkit guidance on hospital caseload thresholds, mean caseload over the preceding 3 years was grouped into four categories using the lower quartile, median, and upper quartile.

### Treatment outcomes

Case managers notify a patient’s status at 12 months to ETS; such ‘outcomes’ were grouped into good, unfavourable, or neutral as per Ditah et al. [8] and Anderson et al. [9] (Additional file 1), except where cases died within 2 weeks of being notified or or where death was considered to be too early to be influenced by the managing clinician or hospital. Good and neutral outcomes were pooled for the regression analysis.

### Analysis

Data were managed and analysed in Stata SE version 13. Age was categorised from a continuous variable into <20, 20–39, 40–64, and 65+ years, guided by known treatment outcome profiles for different age groups [8, 9]. Ethnic group was categorised into White, Black African, Black Other (including Black Caribbean), Indian subcontinent, and Other (e.g. Chinese), as per previous studies [9]. The four social risk factors recorded in ETS (history of past or current homelessness, imprisonment or drug misuse, current alcohol abuse hindering the self-administration of treatment) were compiled into a composite variable with the strata: no or unknown risks, one or more previous, one or more current; current risks override previous risks [10]. PHE centre of notification was grouped into the London PHE centre or elsewhere, in line with previous evidence on the relationship between being treated in London and treatment outcomes [9, 11, 12]. TB notification date was categorised from a continuous variable into pre- and post-toolkit periods (January 2006 to May 2007, June 2007 onwards) [4]. Site of disease was combined with smear status to generate a single variable with three strata: solely extrapulmonary, pulmonary with or without extrapulmonary site(s) and smear negative or smear status missing, and pulmonary with or without extrapulmonary site(s) smear positive. Drug sensitivity at diagnosis was grouped into: sensitive to all first line drugs used as standard treatment in the UK (ethambutol (EMB), isoniazid (INH), pyrazinamide (PZA); by definition in this study all cases were RIF sensitive), resistant to any first line drug, or not culture confirmed. ‘Missing’ here relates to culture-confirmed cases without any information on resistance to EMB, INH, and PZA, as opposed to cases not linked to a culture.

Potential confounders and effect modifiers of the relationship between clinician or hospital caseload and treatment outcomes were identified as per Victora et al. [13]. For the clinician caseload modelling notification date, drug sensitivity, previous diagnosis, social risk factors, and shared clinical management were identified as potential effect modifiers. Sex, age, and shared clinical management were defined as a priori confounders, the latter due to the toolkit’s recommendation of its use for relatively inexperienced clinicians. For the hospital caseload modelling, sex and age were defined as a priori confounders and notification date, age, being UK born (or not), ethnicity, and social risk factors as potential effect modifiers.

Descriptive analyses of the characteristics of the cohort were undertaken. Full random effects logistic regression was the statistical analysis method of choice to account for clustering by clinician for the clinician caseload models and hospital for the hospital caseload models when calculating point estimates, standard errors (SEs) and log likelihoods (clustering structure A). Univariate models were built and contributed to aid the identification of confounders for multivariable modelling. The inclusion of variables in the final multivariable clinician and hospital caseload models depended upon a step-by-step backwards deletion strategy that compared the square roots of estimated mean squared errors and beta coefficients between full (all suggested confounders present) and reduced (deleting one suggested confounder at a time) models, whilst simultaneously allowing for the assessment of confounding and multicollinearity and the maintenance of a priori confounders [14]. Linearity and effect modification were assessed in these models. The estimated intra-cluster correlation coefficient, ρ, was calculated per model and the reliability of each model’s parameter estimate approximations assessed using different numbers of cut points.

### Sensitivity and extended analyses

Analyses were planned to examine the sensitivity of estimates to missing data using the final multivariable regression models derived above. Missing outcome data were recoded to either good/neutral or unfavourable. Missing caseload data were either recoded to above the threshold and placed in a single new clinician or hospital group, or below the threshold and placed in an individual clinician or hospital group per case. Analysis using the caseload of the alternative managing clinician, where one was present, was undertaken. INH resistant cases were excluded from both the hospital and clinician caseload models in light of a recent outbreak of such cases in London. In this outbreak, treatment regimens of 9 or 12 months were recommended, which, if patients changed regimen at the point of culture results, could have resulted in them being recorded as still on treatment- i.e. an unfavourable outcome- at 12 months [15]. Patients with neutral outcomes were excluded as an additional sensitivity analysis for both clinician and hospital caseload. A subgroup analysis of the 10 hospitals for whom further cleaning of the clinician field had been undertaken was planned.

Clustering structure A did not adjust for clustering by hospital for the clinician caseload analysis and by clinician for the hospital caseload analysis. Our data was neither purely hierarchically structured nor were there clean crossed-effects; clinicians frequently managed patients across various different hospitals during the time period studied, often working in multiple hospitals within the same year. This made adjusting for clustering complex. We tested two other clustering structures in sensitivity analyses using multilevel mixed-effects logistic regression with QR decomposition. Structure B assigned all patients managed by a clinician to a single hospital, being the one in which the greatest number of their patients were treated. When a clinician had more than one top hospital, assignment was random between them. This structure assumed that a clinician’s performance was most influenced by the hospital they worked in the most. Structure C split clinician clusters by the hospital in which they worked. This assumed that clinicians perform differently in different hospitals. Analyses using structures A and B were also run restricting to patients with both a reported hospital and managing clinician for the ease of comparison between methodologies.

Further sensitivity analyses were undertaken such that hospital caseload was adjusted for in the final clinician caseload model and vice versa, using both clustering structures B and C.

Additional analyses to explore the impact of choosing other clinician caseload thresholds on the post-toolkit association with unfavourable outcomes (as such an association is more relevant for current policymakers) were also planned. The first explored the impact of very low caseloads (such as might become more common in low burden countries) on the association with treatment outcome, the second utilised decision tree recursive partitioning in JMP 12 (SW) to determine the mean caseload over the past 3 years at which the crude association between clinician caseload and treatment outcome was maximised.

### Ethics, consent, and permissions

This study was approved by the London School of Hygiene MSc Research Ethics Committee (reference 7345). Data were collected and anonymised by PHE, which has National Information Governance Board approval to hold and analyse national surveillance data without informed consent for public health purposes under Section 251 of the National Health Service England Act 2006.

## Results

### TB cases

A total of 67,869 TB cases not diagnosed post-mortem, with treatment expected to last 12 months or less, were notified in England between 2003 and 2012 (Additional file 2). Caseload was calculated as a mean for each year from the preceding 3 years of data, thus cases notified from 2003 to 2005 were utilised to assess caseload for 2006 and could not be included in the analysis. Of the remaining 48,838 cases, 1,468 were missing a treatment outcome, 7,943 a designated treating clinician, and 1,336 a named treating hospital. Table 1 describes the baseline characteristics of these cases, who were generally male, aged 20–39 years of age, had not been previously diagnosed with TB, and had no social risk factors. As documented in previous reports, England has a high proportion of extrapulmonary TB cases compared to some other low-incidence settings [1, 16]. Of all cases, only 3.6 % were resistant to EMB, INH, or PZA (6.1 % of those with a culture result).

**Table 1:** Univariate logistic regression of the association between clinician caseload and treatment outcome

Main exposure/potential confounders/a priori confounders		Overall cohort		Missing 12-month outcome		Unfavourable 12-month outcome		Univariate regression^a^
		No.	Col. %	No.	Row %	No.	Row %	OR (95 % CI)
Overall		48,838	100.0	1,468	3.0	6,777	13.9	–
Clinician caseload^b^								
	10+	24,092	49.3	401	1.7	3,057	12.7	*P* <0.001
	<10	16,803	34.4	767	4.6	2,518	15.0	1.20 (1.10–1.30)
	Missing	7,943	16.3	300	3.8	1,202	15.1	–
Notification date								
	Post-toolkit	39,264	80.4	920	2.3	5,283	13.5	*P* <0.001
	Pre-toolkit	9,574	19.6	548	5.7	1,494	15.6	1.28 (1.19–1.38)
	Missing	0	0.0	0	–	0	–	–
Location								
	Outside London	28,212	57.8	1,338	4.7	4,069	14.4	*P* <0.001
	Inside London	20,618	42.2	130	0.6	2,704	13.1	0.78 (0.70–0.86)
	Missing	8	0.0	0	0.0	4	50.0	–
Sex								
	Male	26,910	55.1	829	3.1	4,051	15.1	*P* <0.001
	Female	21,777	44.6	630	2.9	2,700	12.4	0.80 (0.76–0.85)
	Missing	151	0.3	9	6.0	26	17.2	–
Age, years								
	<20	4,987	10.2	128	2.6	477	9.6	0.66 (0.58-0.75)
	20–39	23,987	49.1	677	2.8	3,334	13.9	*P* <0.001
	40–64	13,534	27.7	400	3.0	1,749	12.9	0.91 (0.85–0.97)
	65+	6,325	13.0	263	4.2	1,215	19.2	1.42 (1.31–1.54)
	Missing	5	0.0	0	0.0	2	40.0	–
Ethnic group								
	White	9,313	19.1	401	4.3	1,580	17.0	*P* <0.001
	Black African	9,666	19.8	243	2.5	1,176	12.2	0.67 (0.61–0.74)
	Black other	1,473	3.0	20	1.4	213	14.5	0.85 (0.71–1.01)
	Indian subcontinent	21,082	43.2	486	2.3	2,753	13.1	0.74 (0.68–0.80)
	Other	5,662	11.6	131	2.3	746	13.2	0.74 (0.67–0.83)
	Missing	1,642	3.4	187	11.4	309	18.8	*–*
UK born								
	No	33,946	69.5	777	2.3	4,517	13.3	*P* <0.001
	Yes	11,946	24.5	397	3.3	1,742	14.6	1.12 (1.05–1.20)
	Missing	2,946	6.0	294	10.0	518	17.6	*–*
Site of disease^c^								
	Extrapulmonary only	21,681	44.4	543	2.5	2,662	12.3	*P* <0.001
	Pulmonary smear negative/unknown	17,483	35.8	578	3.3	2,441	14.0	1.18 (1.10–1.26)
	Pulmonary smear positive	9,399	19.2	313	3.3	1,623	17.3	1.53 (1.41–1.65)
	Missing	275	0.6	34	12.4	51	18.5	*–*
Drug sensitivity								
	Sensitive	26,955	55.2	782	2.9	3,607	13.4	*P* <0.001
	Resistant to INH, EMB, or PZA	1,744	3.6	53	3.0	756	43.3	5.63 (5.01–6.32)
	Not linked to a culture	19,865	40.7	627	3.2	2,364	11.9	0.86 (0.80–0.91)
	Missing	274	0.6	6	2.2	50	18.2	*–*
Previous diagnosis								
	No	39,767	81.4	1,006	2.5	5,227	13.1	*P* <0.001
	Yes	2,941	6.0	96	3.3	494	16.8	1.35 (1.20–1.51)
	Missing	6,130	12.6	366	6.0	1,056	17.2	*–*
Social risk factors^d^								
	No or unknown	47,048	96.3	1,394	3.0	6,352	13.5	*P* <0.001
	One or more previous	829	1.7	19	2.3	144	17.4	1.42 (1.17–1.73)
	One or more current	961	2.0	55	5.7	281	29.2	2.75 (2.34–3.23)
Shared clinical management								
	No or unknown	48,562	99.4	1,457	3.0	6,721	13.8	*P* = 0.02
	Yes	276	0.6	11	4.0	56	20.3	1.45 (1.06–1.98)

Descriptive analysis and univariate random effects logistic regression of the association between clinician caseload and treatment outcome, England, 2006–2012. All regression models adjusted for clustering by clinician

^a^Odds of an unfavourable versus a good or neutral treatment outcome; records missing an outcome excluded

^b^Mean caseload per clinician over the preceding 3 years

^c^Pulmonary site could be with or without extrapulmonary disease

^d^Social risk factors a composite variable of homelessness, imprisonment, drug misuse and alcohol abuse; current risks override previous risks

*CI* Confidence interval, *Col.* Column, *EMB* Ethambutol, *INH* Isoniazid, *OR* Cluster-specific odds ratio, *P*, *P* value, *PZA* Pyrazinamide

### Clinician caseload

**Fig. 1:**
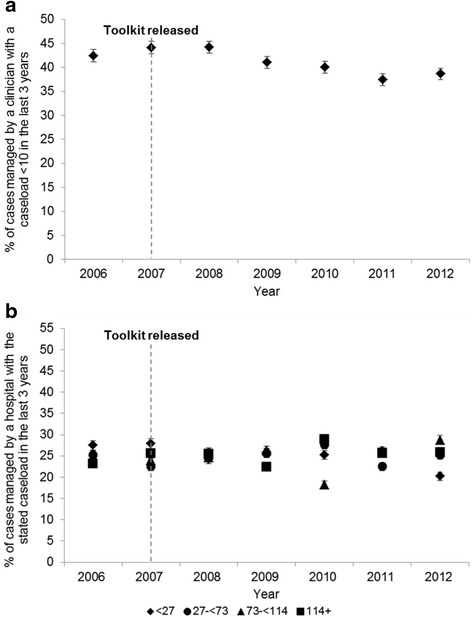
Mean clinician and hospital tuberculosis patient caseloads over the preceding 3 years, England, 2006–2012. Percentage of tuberculosis cases **a** managed by a clinician who saw fewer than 10 cases on average over the preceding 3 years, **b** treated by a hospital with differential quartiles of caseload over the preceding 3 years. Dotted line year toolkit released. Error bars are 95 % confidence intervals

Overall, 2,276 unique clinicians were present using the main groupings and 2,351 with the alternate groupings. Using the former, there was a median of 6 clinicians per hospital across the entire time period (interquartile range, 2–12) and a median caseload of 15 TB cases per clinician over the preceding 3 years (interquartile range, 4–42). Little change in the percentage of cases being managed by clinicians below the toolkit threshold was seen from 2006 to 2012 (42.4 % in 2006, 38.7 % in 2012; Fig. 1a), although calculating caseload for the previous year only (allowing examination of caseload additionally in 2004 and 2005) indicated a drop in the percentage between 2004 and 2006 (51.0 % in 2004, 36.8 % in 2006). Additionally, little redistribution was seen from very low caseloads (less than 2 or between 2 and 5) to higher ones over time (data not shown).

### Clinician caseload and TB case outcomes

Of the 48,838 cases within this cohort, 1,468 (3.0 %) were missing outcome information, 6,777 (13.9 %) had an unfavourable outcome, 942 (1.9 %) a neutral outcome, and the remainder a good outcome (Table 1). Of individuals with an unfavourable outcome, 940 (13.9 %) had died due to the factors listed in Additional file 1, 2,229 (32.9 %) had been lost to follow-up, 3,104 (45.8 %) were still on treatment, and 504 (7.4 %) had had their treatment stopped. Of cases managed by clinicians below the toolkit threshold 15.0 % had an unfavourable outcome versus 12.7 % of cases managed by clinicians above it; 15.6 % of cases in the pre-toolkit period had an unfavourable outcome versus 13.5 % post-toolkit.

There was very strong evidence for clinician caseload being associated with the odds of having an unfavourable outcome in a univariate model; cases managed by clinicians below the threshold had higher cluster-specific odds than those managed by clinicians above it (cluster-specific odds ratio (OR), 1.20; 95 % CI, 1.10–1.30; *P* <0.001; Table 1). Small amounts of within-clinician correlation were observed with very strong evidence (ρ = 0.05; 95 % CI, 0.04–0.07; *P* <0.001); the large number of cases within some clinician groups meant it was necessary to confirm the reliability of each model’s parameter estimate approximations using different numbers of cut points. All potential confounders had evidence of a strong association with the outcome (Table 1). Being female, an ethnicity other than White, or notified within London were associated with lower cluster-specific odds of an unfavourable outcome. Being notified pre-toolkit was associated with higher cluster-specific odds of an unfavourable outcome (likely due to general improvements in care over time), as was shared clinical management.

All potential confounders, aside from drug sensitivity (which was not associated with clinician caseload in a univariate model), were considered for inclusion in the final model using a backwards deletion strategy. At the end of this process, eight remained: notification date, location, sex, age, ethnic group, having previously had TB, social risk factors, and shared clinical management. Including age as a linear variable did not improve model fit. There was very limited statistical evidence for effect modification. In the final multivariable model, ρ was similar to that of the univariate model (0.04; 95 % CI, 0.03–0.05; *P* <0.001) and estimate approximations were again found to be reliable.

The multivariable model also demonstrated very strong evidence for higher cluster-specific odds of an unfavourable outcome being associated with management by clinicians below the caseload threshold (cluster-specific OR, 1.14; 95 % CI, 1.05–1.25; *P* = 0.002; Table 2). There was weak evidence for an association between treatment outcome and shared clinical management (cluster-specific OR, 1.37; 95 % CI, 0.97–1.94; *P* = 0.08).

**Table 2:** Multivariable logistic regression of the association between clinician caseload and treatment outcome

Main exposure/confounders		Multivariable regression^a^
		OR (95 % CI)
Clinician caseload^b^		
	10+	*P* = 0.002
	<10	1.14 (1.05–1.25)
Notification date		
	Post-toolkit	*P* <0.001
	Pre-toolkit	1.33 (1.23–1.45)
Location		
	Outside London	*P* <0.001
	Inside London	0.82 (0.74–0.91)
Sex		
	Male	*P* <0.001
	Female	0.85 (0.80–0.91)
Age, years		
	<20	0.72 (0.63–0.82)
	20–39	*P* <0.001
	40–64	0.88 (0.81–0.95)
	65+	1.30 (1.18–1.44)
Ethnic group		
	White	*P* <0.001
	Black African	0.79 (0.71–0.88)
	Black other	0.88 (0.72–1.07)
	Indian subcontinent	0.86 (0.79–0.94)
	Other	0.86 (0.76–0.97)
Previous diagnosis		
	No	*P* <0.001
	Yes	1.26 (1.12–1.42)
Social risk factors^c^		
	No or unknown	*P* <0.001
	One or more previous	1.44 (1.16–1.77)
	One or more current	2.56 (2.14–3.07)
Shared management		
	No	*P* = 0.08
	Yes	1.37 (0.97–1.94)

Multivariable random effects logistic regression of the association between clinician caseload and treatment outcome, England, 2006–2012. Model adjusted for clustering by clinician and the confounders in the Table; 34,707 records in this model

^a^Odds of an unfavourable versus a good or neutral treatment outcome

^b^Mean caseload per clinician over the preceding 3 years

^c^Social risk factors a composite variable of homelessness, imprisonment, drug misuse, and alcohol abuse; current risks override previous risks

*CI* Confidence interval, *OR* Cluster-specific odds ratio, *P*, *P* value

### Hospital caseload

Although the Department of Health’s toolkit suggests a threshold at the level of the clinician, it may be more appropriate to recommend one at the level of the hospital due to the relative ease of calculation and its ability to better capture the functionality of an entire case management team. Hospital caseload was divided into quartiles: <27, 27–72, 73–113, >114. These figures were conveniently similar to the thresholds proposed by Cegolon et al. [6] and Lake et al. [7]. When hospital caseload was averaged over the last 3 years, little change in the proportion of cases being managed below the toolkit threshold was seen from 2006 to 2012 (Fig. 1b).

### Hospital caseload and TB case outcomes

Overall, 15.9 % of cases treated in a hospital with less than the lower quartile of caseload had an unfavourable outcome versus 12.8–13.4 % in hospitals above it (Table 3). There was very strong evidence for hospital caseload being associated with the cluster-specific odds of having an unfavourable outcome in a univariate model (*P* <0.001; Table 4). With the upper quartile of caseload as the baseline, only the CI of the smallest caseload strata did not cross the null (cluster-specific OR, 1.32; 95 % CI, 1.13–1.55). In this model, there was very strong evidence for weak within-hospital clustering (ρ = 0.03; 95 % CI, 0.02–0.04; *P* <0.001). All potential confounders had evidence of a very strong association with the outcome (Table 4).

**Table 3:** Hospital caseload and treatment outcomes

Main exposure		Overall cohort		Missing 12-month outcome		Unfavourable 12-month outcome	
		No.	Column %	No.	Row %	No.	Row %
Overall		48,838	100.0	1,468	3.0	6,777	13.9
Hospital caseload^a^							
	114+	12,021	24.6	126	1.0	1,564	13.0
	73–113	11,662	23.9	149	1.3	1,563	13.4
	27–72	11,847	24.3	297	2.5	1,520	12.8
	<27	11,972	24.5	717	6.0	1,900	15.9
	Missing	7,943	16.3	300	3.8	1,202	15.1

Descriptive tabulation of hospital caseload and treatment outcome, England, 2006–2012

^a^Mean caseload per hospital over the preceding 3 years

**Table 4:** Univariate and multivariable logistic regression of the association between hospital caseload and treatment outcome

Main exposure/potential confounders/a priori confounders		Univariate regression^a^	Multivariable regression^a^
		OR (95 % CI)	OR (95 % CI)
Hospital caseload^b^			
	114+	*P* <0.001	^e^
	73–113	1.13 (1.00–1.29)	
	27–72	1.06 (0.91–1.24)	
	<27	1.32 (1.13–1.55)	
Notification date			
	Post-toolkit	*P* <0.001	*P* <0.001
	Pre-toolkit	1.25 (1.17–1.33)	1.30 (1.21–1.40)
Location			
	Outside London	*P* <0.001	*P* = 0.16
	Inside London	0.81 (0.71–0.93)	0.90 (0.77–1.04)
Sex			
	Male	*P* <0.001	*P* <0.001
	Female	0.79 (0.75–0.84)	0.85 (0.80–0.90)
Age, years			
	<20	0.67 (0.60–0.75)	0.70 (0.63–0.79)
	20–39	*P* <0.001	*P* <0.001
	40–64	0.93 (0.87–0.99)	0.92 (0.85–0.98)
	65+	1.48 (1.37–1.59)	1.44 (1.32–1.57)
Ethnic group			
	White	*P* <0.001	^e^
	Black African	0.68 (0.63–0.75)	
	Black other	0.88 (0.75–1.03)	
	Indian subcontinent	0.76 (0.71–0.82)	
	Other	0.74 (0.67–0.82)	
UK born			
	No	*P* <0.001	^e^
	Yes	1.10 (1.03–1.17)	
Site of disease^c^			
	Extrapulmonary only	*P* <0.001	–
	Pulmonary smear negative/unknown	1.16 (1.09–1.23)	
	Pulmonary smear positive	1.52 (1.42–1.63)	
Drug sensitivity			
	Sensitive	*P* <0.001	–
	Resistant to INH, EMB, or PZA	5.40 (4.86–6.00)	
	Not linked to a culture	0.87 (0.82–0.92)	
Previous diagnosis			
	No	*P* <0.001	–
	Yes	1.32 (1.19–1.46)	
Social risk factors^d^			
	No or unknown	*P* <0.001	*P* <0.001
	One or more previous	1.33 (1.11–1.60)	1.36 (1.12–1.66)
	One or more current	2.83 (2.44–3.28)	2.67 (2.28–3.13)

Univariate and multivariable random effects logistic regression of the association between hospital caseload and treatment outcome, England, 2006–2012. All regression models adjusted for clustering by hospital; 42,995 cases in the multivariable model, dashed variables not retained

^a^Odds of an unfavourable versus a good or neutral treatment outcome; records missing an outcome excluded

^b^Mean caseload per hospital over the preceding 3 years

^c^Pulmonary site could be with or without extrapulmonary disease

^d^Social risk factors a composite variable of homelessness, imprisonment, drug misuse, and alcohol abuse; current risks override previous risks

^e^Interaction present between hospital caseload, ethnicity and UK born, see Table 5

*CI* Confidence interval, *EMB* Ethambutol, *INH* Isoniazid, *OR* Cluster-specific odds ratio, *p* *P*-value, *PZA* Pyrazinamide

Following the same process as for clinician caseload, a final multivariable model was built for hospital caseload that retained notification date, PHE centre of notification, sex, age, ethnic group, UK born, and social risk factors (Table 4). Interactions were detected between ethnic group (*P* = 0.004) and being UK born (*P* <0.001) and hospital caseload. As ethnic group and being UK born had 10 possible strata combinations between them, they were condensed for the final model into: White UK born, Other UK born, White not UK born, Black not UK born, Indian subcontinent not UK born, Other not UK born. The observed interaction was retained using this categorisation (*P* = 0.01). Among those not born in the UK, the likelihood of an unfavourable outcome may increase with decreased caseload, although CIs generally crossed the null (Table 5). In the UK born, if anything, this pattern was reversed. The association between the combined ethnic group and place of birth variable with treatment outcomes is presented in Table 6; a clear pattern of effect was difficult to discern. In the final model there was very strong evidence for weak within-hospital clustering (ρ = 0.03; 95 % CI, 0.02–0.04; *P* <0.001).

**Table 5:** Multivariable logistic regression of the hospital caseload-treatment outcome association, stratified by country of birth/ethnicity

Main exposure		Multivariable regression^a^ OR (95 % CI)					
		UK born		Not UK born			
		White	Other	White	Black	Indian subcontinent	Other
Hospital caseload^b^	114+	Baseline	Baseline	Baseline	Baseline	Baseline	Baseline
	73–113	1.01 (0.76–1.35)	0.95 (0.73–1.23)	1.16 (0.77–1.74)	1.00 (0.81–1.22)	1.09 (0.92–1.28)	1.14 (0.88–1.49)
	27–72	0.82 (0.61–1.09)	0.68 (0.51–0.91)	1.03 (0.67–1.58)	1.02 (0.81–1.29)	0.95 (0.79–1.15)	1.31 (0.98–1.75)
	<27	0.88 (0.66–1.17)	0.84 (0.61–1.15)	1.35 (0.90–2.02)	1.27 (0.99–1.63)	1.12 (0.91–1.38)	1.47 (1.10–1.96)

Multivariable random effects logistic regression of the association between hospital caseload and treatment outcome stratified by country of birth and ethnicity, England, 2006–2012. Models adjusted for clustering by hospital and for the variables listed in Table 4

^a^Odds of an unfavourable versus a good or neutral treatment outcome; records missing an outcome excluded

^b^Mean caseload per hospital over the preceding 3 years

CI, Confidence interval; OR, Cluster-specific odds ratio

**Table 6:** Multivariable logistic regression of the birth country/ethnicity-treatment outcome association, stratified by hospital caseload

Effect modifier		Multivariable regression^a^ OR (95 % CI)			
		Hospital caseload^b^			
		114+	73–113	27–72	<27
UK born	White	Baseline	Baseline	Baseline	Baseline
	Other	0.91 (0.69–1.20)	0.86 (0.67–1.09)	0.76 (0.60–0.97)	0.87 (0.68–1.12)
Not UK born	White	0.91 (0.64–1.30)	1.04 (0.76–1.43)	1.15 (0.84–1.57)	1.40 (1.08–1.81)
	Black	0.70 (0.55–0.90)	0.69 (0.57–0.85)	0.88 (0.73–1.07)	1.02 (0.85–1.22)
	Indian subcontinent	0.77 (0.61–0.97)	0.82 (0.68–0.99)	0.89 (0.76–1.06)	0.98 (0.85–1.13)
	Other	0.66 (0.50–0.87)	0.75 (0.60–0.94)	1.06 (0.85–1.34)	1.11 (0.91–1.35)

Multivariable random effects logistic regression of the association between country of birth and ethnicity and treatment outcome stratified by hospital caseload, England, 2006–2012. Models adjusted for clustering by hospital and for the variables listed in Table 4

^a^Odds of an unfavourable versus a good or neutral treatment outcome; records missing an outcome excluded

^b^Mean caseload per hospital over the preceding 3 years

*CI* Confidence interval, *OR* Cluster-specific odds ratio

### Sensitivity analysis

The following sensitivity analyses were undertaken: (1) all records with missing outcome information were recoded as having a good/neutral outcome or an unfavourable outcome, (2) all records with missing clinician caseload information were coded to be either above or below the caseload threshold, (3) where present, the alternative named clinician was used to calculate whether a case was managed by an individual above or below the threshold. Analysis (2) was not deemed necessary for the hospital caseload model due to relatively low levels of missingness. Effect estimates were relatively uniform throughout (data not shown), which was especially reassuring given the apparent association between hospital caseload and treatment outcome reporting (Table 3). Exclusion of INH resistant cases also did not appreciably alter effect estimates, nor did the exclusion of patients with neutral treatment outcomes (data not shown).

Encouragingly, an analysis restricted to the 10 hospitals in which we undertook additional clinician name and coding data checks to identify infectious disease or respiratory consultants with greater certainty indicated that the threshold caseload of 10 was still associated with treatment outcome, albeit with low power to test the hypothesis of no association due to the radically reduced size of the dataset (cluster-specific OR, 1.11; 95 % CI, 0.84–1.46; *P* = 0.47; Additional file 3).

Utilising different clustering structures had little impact on the results observed when point estimates and CIs were compared like-with-like in terms of the number of included patients (Additional file 4).

In the clinician caseload model, the inclusion of hospital caseload did little to alter the estimated association between clinician caseload and treatment outcome (cluster-specific OR, 1.10; 95 % CI, 1.01–1.20; *P* = 0.03 with clustering structure B and cluster-specific OR, 1.10; 95 % CI, 1.01–1.21; *P* = 0.03 with structure C). In the hospital caseload model, the inclusion of clinician caseload generally reduced the point estimates observed with both clustering structures (Additional file 5).

### Different thresholds for clinician caseload

In order to explore the relationship post-toolkit between clinician caseload and treatment outcomes at different caseload thresholds, two versions of the final multivariable model were run. The first reduced the caseload threshold to one, as per Gardam et al.’s findings [5], in order to explore the impact of very low average caseloads as might be seen in low-incidence settings. The effect estimate seen was similar to those produced by the 10 case threshold: (cluster-specific OR, 1.13; 95 % CI, 1.01–1.27; *P* = 0.04; Additional file 6a). Recursive partitioning suggested that the caseload threshold creating the strongest association between clinician caseload and treatment outcome was 12.666 cases per year, which was associated with an adjusted OR of 1.15 (95 % CI, 1.04–1.27; *P* = 0.01) post-toolkit (Additional file 6b).

## Discussion

In our study of the relationship between caseloads and treatment outcomes, we observed very strong evidence for higher cluster-specific odds of an unfavourable outcome if TB cases were managed by clinicians who saw less than 10 cases, on average, over the preceding 3 years (cluster-specific OR, 1.14; 95 % CI, 1.05–1.25; *P* = 0.002). Clinician caseload findings were robust when the caseload threshold was reduced further to a mean over 3 years of one. By comparison, the picture with hospital caseload was less clear due to a complex interaction with ethnicity and place of birth; among those not born in the UK, it may be unfavourable to be treated by hospitals with a lower caseload, but these estimates should be taken extremely cautiously.

This is the first study to explore whether the Department of Health’s caseload recommendations for England are linked to TB treatment outcomes. Within our dataset, 16.3 % of patients were missing a named clinician, but including such patients had little impact on effect estimates. The grouping of clinicians using a free-text field may have resulted in misclassification due to spelling errors, common surnames, clinicians moving hospitals, etc. Additionally, some hospitals may have a policy of recording nurses’ names, the names of non-consultants, or assigning patients to a single clinician in ETS. The 2013–2014 Royal College of Physicians’ census identified 1,097 respiratory and 180 infectious disease and tropical medicine consultants for England, Northern Ireland, Scotland and Wales [17], which, even accounting for staff turnover, suggests that we have over-estimated the number of clinicians in our dataset. Such errors are likely to be non-differential, biasing the effect estimate towards the null; we were also reassured to find a similar association in a restricted dataset with additional data checks. By comparison, the treating hospital recorded should be substantially more reliable, although only the last treating hospital is recorded, not where the first critical weeks of treatment are administered. Within the treatment stopped outcome, clinical reasons, such as pregnancy, were included; had these individuals been separable they may have been more appropriately grouped as neutral. HIV status and other comorbidities are unfortunately not collected as part of TB surveillance data. TB case notification is known to be incomplete in England [18], but it is unlikely that missing cases are different in terms of their relationship between caseload and treatment outcome.

We compared a series of different clustering structures in sensitivity analyses, each of which had their specific limitations – structure A failed to account for clustering by hospital within the clinician caseload analysis and vice versa, but was the best methodology for the two main caseload models and maximised the number of analysable patients; structure B made strong assumptions about the relationship between clinicians and hospitals, did not take into account the temporality of a clinician-hospital association, and lost some of the clustering effect of hospital; structure C underestimated the impact of clinician clustering – however, the minimal impact on the point estimates and SEs of each method was reassuring.

Our findings on the association between clinician caseload and treatment outcome are similar to Gardam et al.’s Canadian study [5]. In contrast, the interaction term in our hospital caseload model complicates the comparison to Cegolon et al. [6] and Lake et al. [7]. The observed association between clinician caseload and treatment outcomes is likely more complex than a simple threshold would suggest; clinicians with very large caseloads may be under-resourced, and complex cases may be managed by specialist clinicians who inevitably see very few patients, e.g. paediatricians and experts on HIV-TB co-infection or individuals with social risk factors. Interestingly, we did not see evidence of shared clinical management improving patient outcomes, but the naming of two clinicians may reflect case complexity and may also not capture all instances of dual responsibility. Hospitals with small caseloads may have less experienced clinicians, reduced availability of TB nurses, or less access to Directly Observed Therapy (DOT) workers (use of DOT will have been largely adjusted for in our analysis through proxy variables such as social risk factors, as per National Institute for Health and Care Excellence guidance [19]). At the hospital level, the variability of the association between caseload and treatment outcomes by country of birth and ethnicity may be due to diagnostic delay (and thus the severity of disease at presentation), different sites of disease between subgroups [1, 10], or geographical and socio-economic factors. It should be noted that the increase in the odds of a negative outcome associated with caseload are not as strong as that associated with other key exposures, e.g. social risk factors.

The optimal caseload is expected to be dependent upon the healthcare system in which clinicians and hospitals function and how patients interact with that system. Joined-up multidisciplinary care teams, pathways, and systems with good continuity and oversight [20, 21] ensure that patients have minimal opportunities to become poorly adherent or lost to follow-up, and influence when and how effectively clinicians can engage with their patients. This may be particularly important for patients with comorbidities.

If TB case numbers fell dramatically in England and current patterns of care continued, a higher percentage of cases will be treated by clinicians below the currently recommended threshold, possibly driving a move towards centralised models of care and increasing the need for the national and global sharing of experience. If care is transferred to clinicians or hospitals with larger caseloads, however, issues may arise as expertise is lost in certain geographical areas and patients have to travel further for treatment [7], particularly those requiring DOT. This may result in later and missed diagnoses and create greater barriers to care, which could result in poorer outcomes. Thus, our data suggesting that clinical experience is pertinent even at a very low average clinician caseload threshold is reassuring. It is interesting to note that the effect estimate observed varied little between the tested thresholds. Policymakers should consider all such competing demands when formulating future guidance.

## Conclusions

Although threshold recommendations at the hospital level are likely to be easier to implement and the recording of treating hospital is more likely to be correct than the recording of a named clinician, we recommend that future guidance is based on clinician caseload thresholds. Further research adjusting for the different clinical and nursing specialities involved in case management at the hospital level and exploring such thresholds in greater detail with more reliable clinician name data will be important to strengthen the evidence behind our findings.

### Availability of data and materials

The underlying data in this manuscript arise from a national surveillance dataset and thus cannot be released.

## Additional files

Additional file 1: Treatment outcomes at 12 months. Table of categorised treatment outcomes. (DOCX 21 kb) Additional file 2: Flow chart of data. Flow chart documenting exclusion from the patient cohort. (DOCX 41 kb) Additional file 3: Sensitivity analysis – multivariable random effects logistic regression of the association between clinician caseload and treatment outcomes in a restricted dataset of 10 hospitals. Sensitivity analysis restricted to 4,884 cases from 10 hospitals where the named clinician field was further checked. (DOCX 16 kb) Additional file 4: Sensitivity analysis – multivariable random effects logistic regression of the association between clinician or hospital caseload and treatment outcome, with different clustering structures. Sensitivity analysis of the impact of different clustering structures on the clinician and hospital caseload models. (DOCX 30 kb) Additional file 5: Sensitivity analysis – stratified multivariable random effects logistic regression of the association between hospital caseload and treatment outcome, adjusted for clinician caseload. Sensitivity analysis of the impact of including clinician caseload in the hospital caseload model. (DOCX 20 kb) Additional file 6: Sensitivity analyses – multivariable random effects logistic regression of the association between clinician caseload at different thresholds and treatment outcome, post-toolkit. Sensitivity analyses setting the clinician caseload threshold to one and a level designated by recursive partitioning. (DOCX 24 kb)

## Abbreviations

CI: Confidence interval
DOT: Directly observed therapy
EMB: Ethambutol
ETS: Enhanced Tuberculosis Surveillance
INH: Isoniazid
OR: Odds ratio
PHE: Public Health England
PZA: Pyrazinamide
RIF: Rifampicin
SE: Standard error
TB: Tuberculosis
