# Supplementary material for: Towards better guidance on caseload thresholds to promote positive tuberculosis treatment outcomes: a cohort study
Source: BMC Med. 2016 Mar 23;14:52. doi: 10.1186/s12916-016-0592-8 (PMC4804548; doi:10.1186/s12916-016-0592-8)
Supplement: Additional file 1: — Treatment outcomes at 12 months. Table of categorised treatment outcomes. (DOCX 21 kb) [file 12916_2016_592_MOESM1_ESM.docx]

##### Additional file 1: Treatment outcomes at 12 months

Treatment outcomes from ETS grouped for analysis. Table adapted from Ditah *et al.* and Anderson *et al.* [8;9]. ETS- Enhanced Tuberculosis Surveillance system, TB- tuberculosis

| Outcome recorded in ETS | Definition | Categorised |
| --- | --- | --- |
| Treatment completed | Completed full course of therapy within 12 months of starting treatment / notification. Completion surrogate for cure. | Good |
| Died within two weeks | Patients who died within two weeks of the case being reported or started on treatment, regardless of the cause. | Neutral |
| Died- TB incidental | Patients who died while on treatment for TB or died without starting treatment. TB not cause of death. |  |
| Died- TB caused death | Patients who died while on treatment for TB or died without starting treatment. TB cause of death. | Unfavourable |
| Died- TB contributed | As above, but TB contributed to death. |  |
| Died- cause unknown | As above, but cause of death unknown. |  |
| Lost to follow-up | Patient lost to follow-up before the end of treatment. |  |
| Still on treatment | Patient still on treatment at 12 months. |  |
| Treatment stopped | Patient found to have stopped treatment due to a clinical decision (pregnancy, too ill to continue treatment), of their own volition (but still within the clinical system), or for another not reason listed. |  |
