# Supplementary material for: Towards better guidance on caseload thresholds to promote positive tuberculosis treatment outcomes: a cohort study
Source: BMC Med. 2016 Mar 23;14:52. doi: 10.1186/s12916-016-0592-8 (PMC4804548; doi:10.1186/s12916-016-0592-8)

#### Additional file 2: Flow chart of data

Flow chart documenting the TB cases included in the analysis from the 2003-2012 English cohorts. TB- tuberculosis
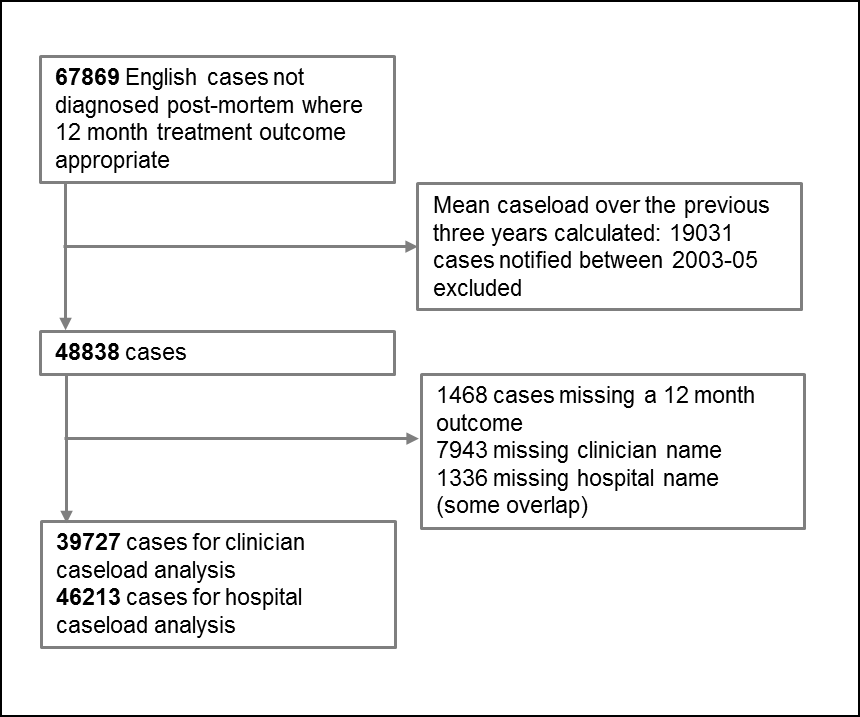

Supplement: Additional file 2: — Flow chart of data. Flow chart documenting exclusion from the patient cohort. (DOCX 41 kb) [file 12916_2016_592_MOESM2_ESM.docx]
