# Supplementary material for: Towards better guidance on caseload thresholds to promote positive tuberculosis treatment outcomes: a cohort study
Source: BMC Med. 2016 Mar 23;14:52. doi: 10.1186/s12916-016-0592-8 (PMC4804548; doi:10.1186/s12916-016-0592-8)
Supplement: Additional file 3: — Sensitivity analysis – multivariable random effects logistic regression of the association between clinician caseload and treatment outcomes in a restricted dataset of 10 hospitals. Sensitivity analysis restricted to 4,884 cases from 10 hospitals where the named clinician field was further checked. (DOCX 16 kb) [file 12916_2016_592_MOESM3_ESM.docx]

#### Additional file 3: Sensitivity analysis- multivariable random effects logistic regression of the association between clinician caseload and treatment outcomes in a restricted dataset of 10 hospitals

Sensitivity analysis restricted to 4,884 cases from 10 hospitals where the named clinician field was further checked. Model adjusted for clustering by clinician and the confounders in the Table. Univariate result (with 5,430 cases) 1.14 (0.88-1.48), 0.32. ^Δ^Odds of having an unfavourable versus a good or neutral treatment outcome. ^Ψ^Mean caseload per clinician over the preceding three years. ‡Social risk factors a composite variable of homelessness, imprisonment, drug misuse and alcohol abuse; current risks override previous risks. CI- confidence interval, OR- cluster-specific odds ratio, p- p-value

| Main exposure/confounders | | Multivariable regression^Δ^ |
| --- | --- | --- |
|  |  | OR (95% CI) |
| Clinician caseload^Ψ^ | |  |
|  | 10+ | p=0.47 |
|  | <10 | 1.11 (0.84-1.46) |
| Notification date |  |  |
|  | post-toolkit | p<0.001 |
|  | pre-toolkit | 1.74 (1.40-2.16) |
| Location |  |  |
|  | Outside London | p=0.94 |
|  | Inside London | 1.01 (0.71-1.44) |
| Gender |  |  |
|  | Male | p=0.35 |
|  | Female | 0.92 (0.76-1.10) |
| Age (years) | |  |
|  | <20 | 0.80 (0.56-1.15) |
|  | 20-<40 | p=0.03 |
|  | 40-<65 | 0.86 (0.70-1.07) |
|  | 65+ | 1.39 (1.02-1.90) |
| Ethnic group | |  |
|  | White | p=0.05 |
|  | Black African | 0.65 (0.48-0.89) |
|  | Black other | 0.91 (0.59-1.40) |
|  | Indian subcontinent | 0.76 (0.57-1.02) |
|  | Other | 0.65 (0.46-0.92) |
| Previous diagnosis |  |  |
|  | No | p=0.49 |
|  | Yes | 0.85 (0.54-1.34) |
| Social risk factors‡ | |  |
|  | No or unknown | p<0.001 |
|  | One or more previous | 1.32 (0.74-2.37) |
|  | One or more current | 3.54 (2.24-5.59) |
| Shared management | |  |
|  | No | p=0.67 |
|  | Yes | 1.67 (0.16-17.27) |
