# Supplementary material for: Towards better guidance on caseload thresholds to promote positive tuberculosis treatment outcomes: a cohort study
Source: BMC Med. 2016 Mar 23;14:52. doi: 10.1186/s12916-016-0592-8 (PMC4804548; doi:10.1186/s12916-016-0592-8)
Supplement: Additional file 4: — Sensitivity analysis – multivariable random effects logistic regression of the association between clinician or hospital caseload and treatment outcome, with different clustering structures. Sensitivity analysis of the impact of different clustering structures on the clinician and hospital caseload models. (DOCX 30 kb) [file 12916_2016_592_MOESM4_ESM.docx]

#### Additional file 4: Sensitivity analysis- multivariable random effects logistic regression of the association between clinician or hospital caseload and treatment outcome, with different clustering structures

Sensitivity analysis of different clustering structures on the outputs of the clinician caseload a) univariate and b) multivariable models or the hospital caseload c) univariate and d) multivariable models. Structure A (clinician clustering alone adjusted for in the clinician caseload models, hospital clustering alone adjusted for in the hospital caseload models) is presented in the main analysis Tables (1, 2, 4 and 5). Structure A no missing data- as Structure A, but restricted to records where both a managing clinician and hospital are named. Structure B- clinicians assigned to the hospital which had the maximum number of their patients. Structure B no missing data- as Structure B, but restricted to records where both a managing clinician and hospital are named. Structure C- clinician clusters split by hospital; this structure automatically contains only records where both the managing clinician and hospital were recorded. Multivariable clinician caseload models adjusted for the variables listed in Table 2. Multivariable hospital caseload models adjusted for the variables listed in Table 4 and presented stratified by ethnicity and country of birth. ^Ψ^Mean caseload per clinician/hospital over the preceding three years. ^Δ^Odds of an unfavourable versus a good or neutral treatment outcome; records missing an outcome excluded. CI- confidence interval, OR- cluster-specific odds ratio

**a)**

| Clustering structure/clinician caseload^Ψ^ | | Univariate regression^Δ^ |
| --- | --- | --- |
|  |  | OR (95% CI) |
| A no missing data | |  |
|  | 10+ | p<0.001 |
|  | <10 | 1.18 (1.09-1.29) |
| B | |  |
|  | 10+ | p=0.001 |
|  | <10 | 1.15 (1.06-1.25) |
| B no missing data |  |  |
|  | 10+ | p=0.002 |
|  | <10 | 1.14 (1.05-1.23) |
| C (no missing data) | |  |
|  | 10+ | p=0.004 |
|  | <10 | 1.13 (1.04-1.22) |

**b)**

| Clustering structure/clinician caseload^Ψ^ | | Multivariable regression^Δ^ |
| --- | --- | --- |
|  |  | OR (95% CI) |
| A no missing data | |  |
|  | 10+ | p=0.01 |
|  | <10 | 1.13 (1.04-1.24) |
| B | |  |
|  | 10+ | p=0.01 |
|  | <10 | 1.12 (1.03-1.22) |
| B no missing data |  |  |
|  | 10+ | p=0.02 |
|  | <10 | 1.11 (1.02-1.21) |
| C (no missing data) | |  |
|  | 10+ | p=0.02 |
|  | <10 | 1.11 (1.02-1.21) |

**c)**

| Clustering structure/hospital caseload^Ψ^ | | Univariate regression^Δ^ |
| --- | --- | --- |
|  |  | OR (95% CI) |
| A no missing data | |  |
|  | 114+ | p=0.001 |
|  | 73-<114 | 1.01 (0.87-1.18) |
|  | 27-<73 | 0.95 (0.80-1.12) |
|  | <27 | 1.21 (1.02-1.43) |
| B | |  |
|  | 114+ | p<0.001 |
|  | 73-<114 | 1.02 (0.89-1.18) |
|  | 27-<73 | 0.97 (0.83-1.14) |
|  | <27 | 1.24 (1.06-1.44) |
| B no missing data |  |  |
|  | 114+ | p<0.001 |
|  | 73-<114 | 1.02 (0.89-1.18) |
|  | 27-<73 | 0.97 (0.83-1.14) |
|  | <27 | 1.24 (1.06-1.44) |
| C (no missing data) | |  |
|  | 114+ | p<0.001 |
|  | 73-<114 | 1.02 (0.87-1.19) |
|  | 27-<73 | 0.99 (0.83-1.17) |
|  | <27 | 1.24 (1.05-1.47) |

**d)**

| Clustering structure/hospital caseload^Ψ^ | | Multivariable regression^Δ^ OR (95% CI) | | | | | |
| --- | --- | --- | --- | --- | --- | --- | --- |
|  |  | UK born | | Not UK born | | | |
|  |  | White | Other | White | Black | Indian subcontinent | Other |
| A no missing data | 114+ | baseline | baseline | Baseline | baseline | baseline | baseline |
|  | 73-<114 | 0.94 (0.68-1.29) | 0.79 (0.59-1.05) | 0.99 (0.63-1.56) | 0.87 (0.69-1.10) | 1.02 (0.85-1.22) | 0.98 (0.72-1.33) |
|  | 27-<73 | 0.73 (0.53-1.00) | 0.57 (0.42-0.78) | 0.74 (0.46-1.19) | 0.92 (0.71-1.19) | 0.89 (0.73-1.10) | 1.08 (0.78-1.50) |
|  | <27 | 0.78 (0.57-1.06) | 0.67 (0.47-0.94) | 1.08 (0.70-1.68) | 1.16 (0.88-1.53) | 1.04 (0.83-1.30) | 1.26 (0.92-1.74) |
| B | 114+ | baseline | baseline | Baseline | baseline | baseline | baseline |
|  | 73-<114 | 0.96 (0.70-1.32) | 0.83 (0.62-1.10) | 1.01 (0.64-1.59) | 0.91 (0.72-1.15) | 1.05 (0.87-1.25) | 1.02 (0.76-1.38) |
|  | 27-<73 | 0.77 (0.56-1.05) | 0.62 (0.46-0.84) | 0.78 (0.49-1.25) | 0.98 (0.76-1.25) | 0.95 (0.78-1.15) | 1.16 (0.84-1.61) |
|  | <27 | 0.82 (0.61-1.10) | 0.72 (0.52-1.01) | 1.15 (0.75-1.76) | 1.22 (0.94-1.58) | 1.11 (0.90-1.37) | 1.34 (0.98-1.82) |
| B no missing data | 114+ | baseline | baseline | Baseline | baseline | baseline | baseline |
|  | 73-<114 | 0.96 (0.70-1.32) | 0.83 (0.62-1.10) | 1.01 (0.64-1.59) | 0.91 (0.72-1.15) | 1.05 (0.87-1.25) | 1.02 (0.76-1.38) |
|  | 27-<73 | 0.77 (0.56-1.05) | 0.62 (0.46-0.84) | 0.78 (0.49-1.25) | 0.98 (0.76-1.25) | 0.95 (0.78-1.15) | 1.16 (0.84-1.61) |
|  | <27 | 0.82 (0.61-1.10) | 0.72 (0.52-1.01) | 1.15 (0.75-1.76) | 1.22 (0.94-1.58) | 1.11 (0.90-1.37) | 1.34 (0.98-1.82) |
| C (no missing data) | 114+ | baseline | baseline | Baseline | baseline | baseline | baseline |
|  | 73-<114 | 0.94 (0.68-1.29) | 0.82 (0.61-1.09) | 0.99 (0.63-1.57) | 0.89 (0.70-1.13) | 1.03 (0.85-1.24) | 1.00 (0.73-1.35) |
|  | 27-<73 | 0.75 (0.55-1.04) | 0.61 (0.45-0.84) | 0.76 (0.47-1.23) | 0.96 (0.74-1.25) | 0.93 (0.75-1.15) | 1.13 (0.81-1.58) |
|  | <27 | 0.79 (0.58-1.08) | 0.70 (0.50-1.00) | 1.11 (0.71-1.72) | 1.19 (0.90-1.57) | 1.08 (0.86-1.36) | 1.29 (0.94-1.78) |
