# Supplementary material for: Towards better guidance on caseload thresholds to promote positive tuberculosis treatment outcomes: a cohort study
Source: BMC Med. 2016 Mar 23;14:52. doi: 10.1186/s12916-016-0592-8 (PMC4804548; doi:10.1186/s12916-016-0592-8)
Supplement: Additional file 5: — Sensitivity analysis – stratified multivariable random effects logistic regression of the association between hospital caseload and treatment outcome, adjusted for clinician caseload. Sensitivity analysis of the impact of including clinician caseload in the hospital caseload model. (DOCX 20 kb) [file 12916_2016_592_MOESM5_ESM.docx]

#### Additional file 5: Sensitivity analysis- stratified multivariable random effects logistic regression of the association between hospital caseload and treatment outcome, adjusted for clinician caseload

Sensitivity analysis of the impact of including clinician caseload in the hospital caseload model. Models adjusted for clustering by hospital and clinician using a) structure B (clinicians assigned to the hospital which had the maximum number of their patients) or b) structure C (clinician clusters split by hospital) and for the variables listed in Table 4. ^Δ^Odds of an unfavourable versus a good or neutral treatment outcome; records missing an outcome excluded. ^Ψ^Mean caseload per hospital over the preceding three years. CI- confidence interval, OR- cluster-specific odds ratio

**a)**

| Main exposure | | Multivariable regression^Δ^ (OR (95% CI)) | | | | | |
| --- | --- | --- | --- | --- | --- | --- | --- |
|  |  | UK born | | Not UK born | | | |
|  |  | White | Other | White | Black | Indian subcontinent | Other |
| Hospital caseload^Ψ^ | 114+ | baseline | baseline | baseline | baseline | baseline | baseline |
|  | 73-<114 | 0.96 (0.70-1.32) | 0.83 (0.62-1.10) | 1.01 (0.64-1.58) | 0.92 (0.73-1.15) | 1.05 (0.88-1.25) | 1.03 (0.76-1.39) |
|  | 27-<73 | 0.76 (0.56-1.04) | 0.61 (0.45-0.83) | 0.77 (0.48-1.24) | 0.97 (0.76-1.24) | 0.94 (0.77-1.14) | 1.16 (0.84-1.60) |
|  | <27 | 0.79 (0.59-1.07) | 0.71 (0.51-0.99) | 1.11 (0.72-1.70) | 1.19 (0.92-1.54) | 1.08 (0.88-1.33) | 1.30 (0.95-1.77) |

**b)**

| Main exposure | | Multivariable regression^Δ^ (OR (95% CI)) | | | | | |
| --- | --- | --- | --- | --- | --- | --- | --- |
|  |  | UK born | | Not UK born | | | |
|  |  | White | Other | White | Black | Indian subcontinent | Other |
| Hospital caseload^Ψ^ | 114+ | baseline | baseline | baseline | baseline | baseline | baseline |
|  | 73-<114 | 0.94 (0.68-1.30) | 0.82 (0.61-1.09) | 0.99 (0.63-1.56) | 0.89 (0.70-1.13) | 1.03 (0.85-1.24) | 1.00 (0.74-1.36) |
|  | 27-<73 | 0.75 (0.54-1.03) | 0.61 (0.44-0.83) | 0.75 (0.47-1.21) | 0.95 (0.74-1.24) | 0.92 (0.75-1.14) | 1.13 (0.81-1.57) |
|  | <27 | 0.77 (0.56-1.05) | 0.69 (0.49-0.97) | 1.07 (0.69-1.66) | 1.16 (0.88-1.52) | 1.05 (0.84-1.32) | 1.25 (0.91-1.73) |
