# Supplementary material for: Towards better guidance on caseload thresholds to promote positive tuberculosis treatment outcomes: a cohort study
Source: BMC Med. 2016 Mar 23;14:52. doi: 10.1186/s12916-016-0592-8 (PMC4804548; doi:10.1186/s12916-016-0592-8)
Supplement: Additional file 6: — Sensitivity analyses – multivariable random effects logistic regression of the association between clinician caseload at different thresholds and treatment outcome, post-toolkit. Sensitivity analyses setting the clinician caseload threshold to one and a level designated by recursive partitioning. (DOCX 24 kb) [file 12916_2016_592_MOESM6_ESM.docx]

#### Additional file 6: Sensitivity analyses- multivariable random effects logistic regression of the association between clinician caseload at different thresholds and treatment outcome, post-toolkit

Sensitivity analyses setting the clinician caseload threshold to a) one and b) 12.666, as determined by recursive partitioning. Post-toolkit cases only; 28,718 cases in model. Model adjusted for clustering by clinician and the confounders in the Table. ^Δ^Odds of having an unfavourable versus a good or neutral treatment outcome. ^Ψ^Mean caseload per clinician over the preceding three years. ‡Social risk factors a composite variable of homelessness, imprisonment, drug misuse and alcohol abuse; current risks override previous risks. CI- confidence interval, OR- cluster-specific odds ratio, p- p-value

**a)**

| Main exposure/confounders | | Multivariable regression^Δ^ |
| --- | --- | --- |
|  |  | OR (95% CI) |
| Clinician caseload^Ψ^ | |  |
|  | 1+ | p=0.04 |
|  | <1 | 1.13 (1.01-1.27) |
| Location |  |  |
|  | Outside London | p<0.001 |
|  | Inside London | 0.81 (0.72-0.92) |
| Gender |  |  |
|  | Male | p<0.001 |
|  | Female | 0.85 (0.79-0.92) |
| Age (years) | |  |
|  | <20 | 0.69 (0.60-0.80) |
|  | 20-<40 | p<0.001 |
|  | 40-<65 | 0.87 (0.80-0.95) |
|  | 65+ | 1.28 (1.15-1.43) |
| Ethnic group | |  |
|  | White | p=0.04 |
|  | Black African | 0.84 (0.74-0.95) |
|  | Black other | 0.88 (0.70-1.10) |
|  | Indian subcontinent | 0.87 (0.79-0.96) |
|  | Other | 0.86 (0.75-0.99) |
| Previous diagnosis |  |  |
|  | No | p<0.001 |
|  | Yes | 1.29 (1.13-1.46) |
| Social risk factors‡ | |  |
|  | No or unknown | p<0.001 |
|  | One or more previous | 1.42 (1.14-1.76) |
|  | One or more current | 2.54 (2.10-3.07) |
| Shared management | |  |
|  | No | p=0.01 |
|  | Yes | 1.64 (1.12-2.40) |

**b)**

| Main exposure/confounders | | Multivariable regression^Δ^ |
| --- | --- | --- |
|  |  | OR (95% CI) |
| Clinician caseload^Ψ^ | |  |
|  | 12.666+ | p=0.01 |
|  | <12.666 | 1.15 (1.04-1.27) |
| Location |  |  |
|  | Outside London | p<0.001 |
|  | Inside London | 0.83 (0.74-0.94) |
| Gender |  |  |
|  | Male | p<0.001 |
|  | Female | 0.85 (0.79-0.92) |
| Age (years) | |  |
|  | <20 | 0.69 (0.59-0.80) |
|  | 20-<40 | p<0.001 |
|  | 40-<65 | 0.87 (0.80-0.95) |
|  | 65+ | 1.28 (1.15-1.43) |
| Ethnic group | |  |
|  | White | p=0.05 |
|  | Black African | 0.84 (0.74-0.95) |
|  | Black other | 0.88 (0.70-1.10) |
|  | Indian subcontinent | 0.88 (0.80-0.97) |
|  | Other | 0.86 (0.75-0.99) |
| Previous diagnosis |  |  |
|  | No | p<0.001 |
|  | Yes | 1.29 (1.13-1.46) |
| Social risk factors‡ | |  |
|  | No or unknown | p<0.001 |
|  | One or more previous | 1.42 (1.15-1.76) |
|  | One or more current | 2.54 (2.10-3.07) |
| Shared management | |  |
|  | No | p=0.01 |
|  | Yes | 1.64 (1.12-2.41) |
